# Identification of co-expressed central genes and transcription factors in acute myocardial infarction and diabetic nephropathy

**DOI:** 10.1186/s12920-024-01906-7

**Published:** 2024-05-20

**Authors:** Bo Li, Xu Zhao, Wanrun Xie, Zhenzhen Hong, Ye Cao, Yi Zhang, Yan Ding

**Affiliations:** 1 Department of Endocrinology, Quanzhou First Hospital Affiliated to Fujian Medical University, Quanzhou, 362000 Fujian China; 2 Emergency and Critical Care Center, Renmin Hospital, Hubei University of Medicine, No. 37 Chaoyang Middle Road, Shiyan, 442000 Hubei China; 3 Department of Cardiology, Fujian Provincial Hospital, Shengli Clinical Medical College of Fujian Medical University, Fuzhou, 350001 Fujian China; 4 Department of Cardiology, Renmin Hospital, Hubei University of Medicine, No. 37 Chaoyang Middle Road, Shiyan, 442000 Hubei China; 5 Hubei Key Laboratory of Embryonic Stem Cell Research, Hubei Provincial Clinical Research Center for Umbilical Cord Blood Hematopoietic Stem Cells, Taihe Hospital, Hubei University of Medicine, Shiyan, 442000 Hubei China

**Keywords:** Acute myocardial infarction, Diabetic nephropathy, Bioinformatics, Differentially expressed genes, Hub genes, Transcription factor

## Abstract

**Background:**

Acute myocardial infarction (AMI) and diabetic nephropathy (DN) are common clinical co-morbidities, but they are challenging to manage and have poor prognoses. There is no research on the bioinformatics mechanisms of comorbidity, and this study aims to investigate such mechanisms.

**Methods:**

We downloaded the AMI data (GSE66360) and DN datasets (GSE30528 and GSE30529) from the Gene Expression Omnibus (GEO) platform. The GSE66360 dataset was divided into two parts: the training set and the validation set, and GSE30529 was used as the training set and GSE30528 as the validation set. After identifying the common differentially expressed genes (DEGs) in AMI and DN in the training set, gene ontology (GO) and Kyoto Encyclopedia of Genes and Genomes (KEGG) enrichment analyses and protein–protein interaction (PPI) network construction were performed. A sub-network graph was constructed by MCODE, and 15 hub genes were screened by the Cytohubba plugin. The screened hub genes were validated, and the 15 screened hub genes were subjected to GO, KEGG, Gene MANIA analysis, and transcription factor (TF) prediction. Finally, we performed TF differential analysis, enrichment analysis, and TF and gene regulatory network construction.

**Results:**

A total of 46 genes (43 up-regulated and 3 down-regulated) were identified for subsequent analysis. GO functional analysis emphasized the presence of genes mainly in the vesicle membrane and secretory granule membrane involved in antigen processing and presentation, lipopeptide binding, NAD + nucleosidase activity, and Toll-like receptor binding. The KEGG pathways analyzed were mainly in the phagosome, neutrophil extracellular trap formation, natural killer cell-mediated cytotoxicity, apoptosis, Fc gamma R-mediated phagocytosis, and Toll-like receptor signaling pathways. Eight co-expressed hub genes were identified and validated, namely TLR2, FCER1G, CD163, CTSS, CLEC4A, IGSF6, NCF2, and MS4A6A. Three transcription factors were identified and validated in AMI, namely NFKB1, HIF1A, and SPI1.

**Conclusions:**

Our study reveals the common pathogenesis of AMI and DN. These common pathways and hub genes may provide new ideas for further mechanistic studies.

**Supplementary Information:**

The online version contains supplementary material available at 10.1186/s12920-024-01906-7.

## Introduction

The heart and kidneys are vital organs in the body for maintaining circulatory and internal environmental homeostasis. There is cross-talk between the functions of the two organs, and acute or chronic injury to either organ may cause adaptive changes or adverse reactions in the two organs, resulting in cardiorenal syndrome [1]. Acute myocardial infarction (AMI) is also one of the most common types of acute kidney injury. While diabetes is one of the major risk factors for atherosclerotic vascular disease, it promotes circulatory disorders in the renal and coronary arteries, often concurrently or serially, causing CHD or diabetic nephropathy (DN). Cardiovascular disease mediates nearly 50–80% of deaths in patients with diabetes [2, 3], while diabetic nephropathy is a widespread complication of diabetes and a major cause of end-stage renal disease (ESRD) [4]. Although AMI and DN are both circulatory disorders with common co-morbidities, the molecular mechanisms explaining the coexistence of these two diseases are unclear. Studying common transcriptional signatures may provide new insights into the common pathogenesis of AMI and DN.

AMI is inextricably associated with DN, and studies have shown that the 10-year risk of death is also increased by 10% for mild renal impairment compared to normal renal function in AMI patients. Severe renal impairment has more than doubled the risk of death at 1 year after AMI, and this increased risk persisted, with creatinine clearance becoming a risk factor beyond other risk factors and second only to age by 10 years [5]. Emergency percutaneous coronary intervention (PCI) is widely acknowledged as an effective treatment for AMI to save the endangered myocardium and reduce mortality and disability. However, contrast-induced nephropathy (CIN) is a common complication in patients with PCI, and hyperglycemia increases the risk of CIN after PCI [6], is associated with a lengthened hospital stay and later adverse outcomes [7, 8], and is associated with a significantly increased risk of CIN in AMI patients undergoing emergency PCI [9, 10], which is more pronounced in patients with DN. Diabetes increases the risk of macrovascular and microvascular complications, including AMI and DN. Studies have shown that the annual cases of AMI and ESRD in Singaporean diabetic patients are projected to increase by 50% by 2050 [11, 12], and it is assumed that this risk may exist in many countries around the world. It is urgent to analyze the co-morbidity mechanisms of AMI and DN from a bioinformatics perspective, which is why we conducted the study.

## Materials and methods

### Data source

**Fig. 1:**
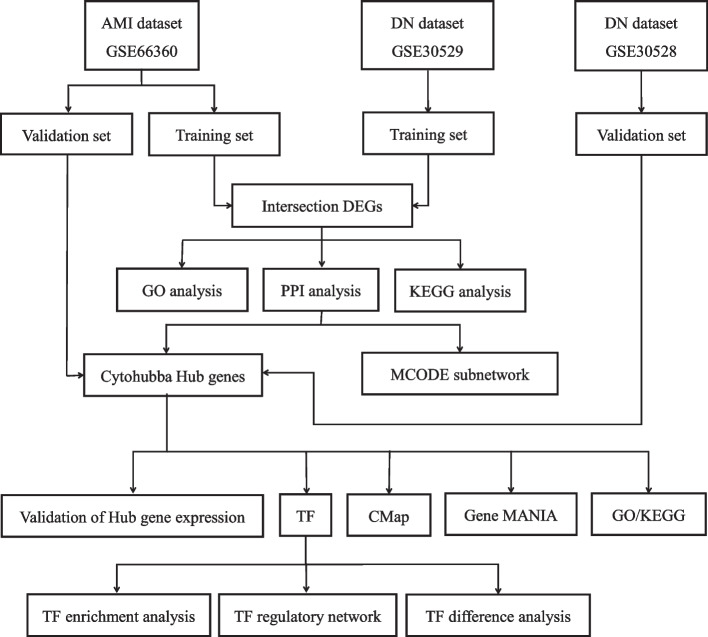
Study flow chart

We downloaded the expression profile dataset of endothelial cells isolated from the circulation of AMI patients (GSE66360), the transcriptome dataset of glomerular specimens from DN patients (GSE30528), and the transcriptome dataset of tubular specimens from DN patients (GSE30529) from the GEO (http://www.ncbi.nlm.nih.gov/geo/) database. GSE66360 includes endothelial cells isolated from the circulation of 49 AMI and 50 healthy controls; we selected the first 13 controls and 21 AMI samples as the training set and the last 37 controls and 28 AMI samples as the validation set. GSE30528 includes transcriptomic data from glomerular specimens of 13 controls and 9 DNs as the validation set. GSE30529 includes 12 controls and 10 DNs of renal tubular transcriptome data as the training set. The GSE30528 and GSE30529 datasets were matched for gender, and while the difference in age was significant in GSE30528 (*p* = 3.2E-02), the difference in gender was not in GSE30529 (*p* = 0.149). The age range for both sexes in GSE66360 was consistent. The flow chart of this paper is shown in Fig. 1.

### Differentially expressed genes

We cleaned the data, removed the probes without gene annotation, averaged the duplicate probes, normalized and log2-transformed the data, and performed difference analysis for the GSE66360 training set and GSE30529 using the "limma" R package [13]. DEGs were screened based on an adjusted *p*-value of 0.05 and a |log FC| of > 0.585. The "pheatmap" R package and the "ggplot2" R package were used for volcano plotting and heat map plotting, respectively. Then, we used the "VennDiagram" R package to generate Venn diagrams of up-and-down-regulated genes for the intersecting DEGs in both datasets. Due to the batch effect, different datasets cannot be merged directly, so in our study, we used the fetch intersection method to enhance the robustness of DEG analysis. After removing genes with opposite expression trends, we obtained common DEGs for the two diseases.

### Enrichment analyses of common DEGs

GO functional enrichment analysis mainly included three aspects: gene biological process (BP), cellular component (CC), and molecular function (MF). KEGG pathway enrichment is the integration of gene function and genomics as a holistic network to study gene function annotation and gene pathway enrichment. Common DEGs were submitted to enrichment analysis with an adjusted *p*-value of 0.05 as a screening criterion using the clusterProfiler package [14]. Results are presented using the Ggplot2 package (https://ggplot2.tidyverse.org).

### PPI network graph construction, model analysis, and candidate gene screening

The PPI network was constructed by applying the String [15] database (http://string-db.org) with a minimum required interaction score of medium confidence 0.4 as the screening criterion and hiding disconnected nodes in the network. The network information was imported into Cytoscape [16] for visualization, the sub-network maps were constructed by the MCODE module, and the top 15 candidate core genes were screened by the Cytohubba plugin's Degree module. CytoHubba is a tool designed to assist in network topology analysis. Its degree algorithm works by calculating the number of connections that exist between nodes. The more connections a node has in the network, the higher the degree of the node, and proteins with a high degree of degree are more likely to be key proteins.

### GO, KEGG, and GeneMANIA analyses of candidate core genes

We performed GO and KEGG analyses of the top 15 candidate core genes screened by the Cytohubba plugin using a *p*-value of 0.05 as the filtering criterion, and GeneMANIA (http://www.genemania.org/) was used for candidate core gene correlation gene analysis [17].

### Connectivity map (CMap) analysis of candidate core genes

The CMap [18] database online resource (https://clue.io) was used to identify drug candidates. This gene expression database uses the gene-expression differences in human cells after treatment with different disruptors to create a database of biological applications where disruptor, gene expression, and disease are interrelated. We submitted the previously screened top 15 candidate core genes to the CMAP website for small molecule drugs that may improve the prognosis of AMI and DN comorbidity. The correlation score between drugs and DEGs is expressed on a scale of -100 to 100, with negative values representing interfering gene expression patterns, indicating that this interference has potential therapeutic effects. We selected < -80 as the cutoff value to screen drug candidates and selected the top 10 to be displayed.

### Prediction and verification of transcription factors

We performed TF enrichment analysis of candidate core genes using TRRUST version 2 online software (https://www.grnpedia.org/trrust/) and imported the obtained TF enrichment results into Cytoscape to obtain the regulatory network maps of TFs and genes. We used the limma package for differential analysis of TF and visualization of TF expression data in the GSE66360 training set and GSE30528 dataset.

### Validation and analysis of hub genes

Both external datasets, GSE66360 (AMI) validation set and GSE30528 (DN), were used to validate the expression of candidate hub genes. The data were analyzed using the limma package, the data were log2 processed and corrected, the data were compared between groups with a *p*-value of < 0.05 as the criterion for significant differences, and the visualization of the validation results was performed with the Ggpubr package. Candidate genes that passed validation were considered pivotal genes.

### Evaluation of immune infiltrating cells

The fraction of co-expressed genes in AMI circulating endothelial cells and DN renal tubular cells in 28 immune cell types was evaluated separately using the single sample gene set enrichment analysis (ssGSEA) method. The ssGSEA analysis was completed using the "GSVA" and "GSEABase" packages in R, and the results were visualized using the ggplot2 package. The ssGSEA is a widely used technique for analyzing immune cell infiltration, where it uses gene expression data to compare each sample to a specific set of immune cell genes. This comparison results in an immune cell content score, which is calculated using the GSVA function. The higher the score, the higher the immune cell content in the sample. The scoring and grouping data are then combined and corrected, and a heat map is generated as the final output.

## Results

### Identification of DEGs

**Fig. 2:**
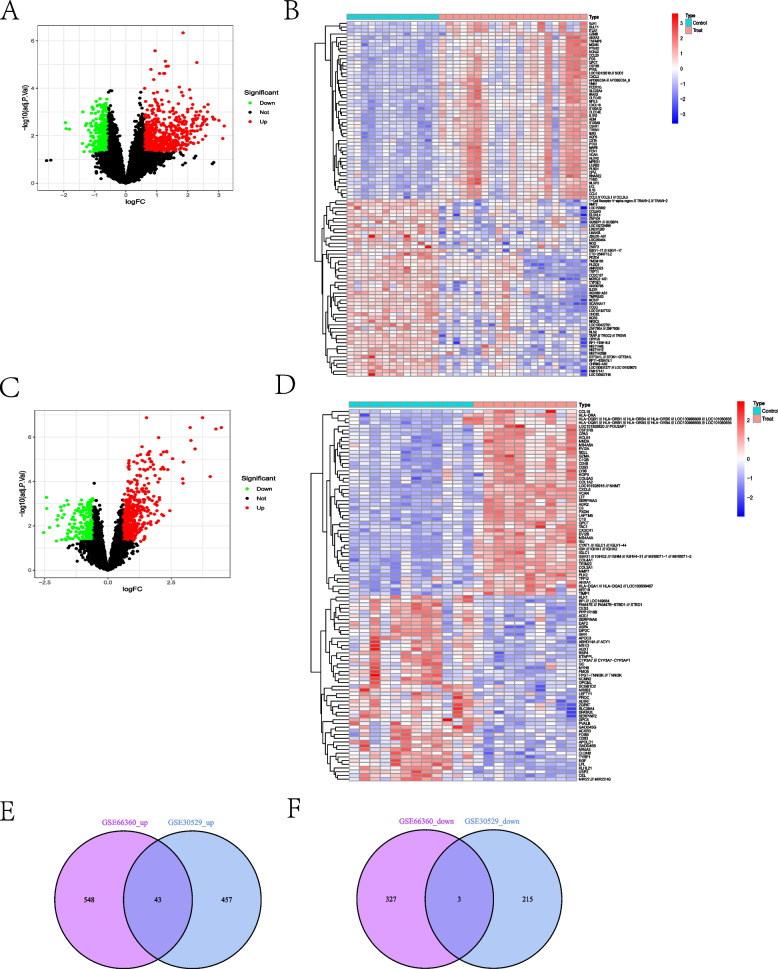
Volcano and heat plots for difference analysis and co-morbidity intersection Venn diagrams. Heat plot and volcano plot for difference analysis of the GSE66360 training set (**A**, **B**); heat plot and volcano plot for difference analysis of the GSE30529 data set (**C**, **D**); Venn plots of up-regulated DEG intersections and Venn plots of down-regulated DEG intersections for the GSE66360 training set and the GSE30529 data set (**E**, **F**)

Difference analysis and visualization of the GSE66360 training set and the GSE30529 dataset were performed using an adjusted *p*-value of 0.05 and a |log FC| of > 0.585 as filtering criteria (Fig. 2A–D), and 922 DEGs were obtained in the GSE66360 validation set and 719 differential genes in the GSE30529. A total of 43 up-regulated genes and 3 down-regulated genes were obtained after taking the intersection of the two disease DEGs (Fig. 2E, F).

### GO and KEGG enrichment analysis of common DEGs

**Fig. 3:**
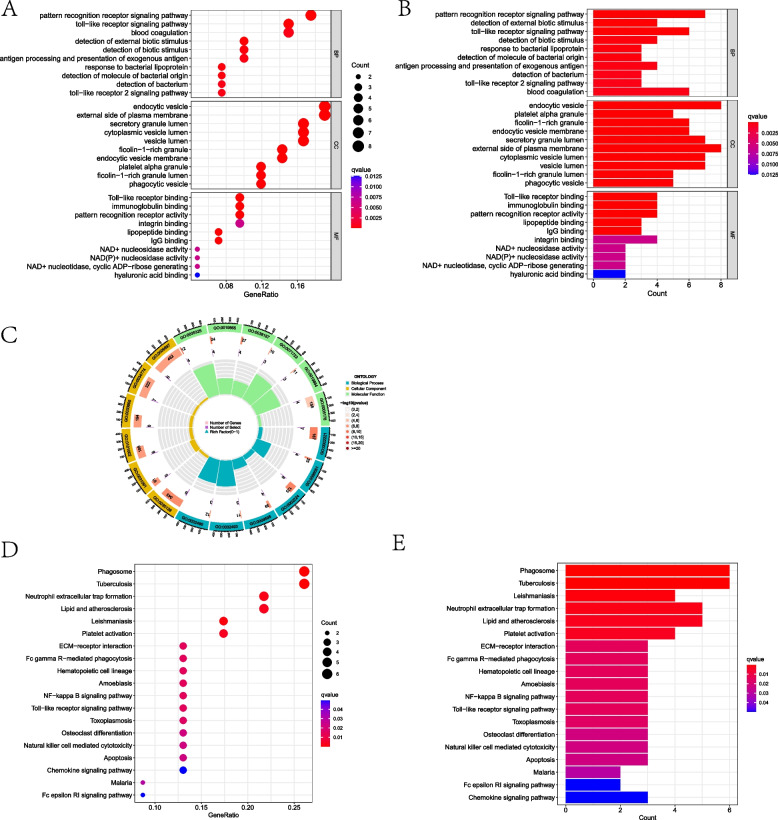
Bar and bubble plots of GO/KEGG enrichment analysis of common DEGs and circle plots of GO analysis; GO functional analysis of common DEGs (**A**, **B**); circle plots of GO analysis (**C**); KEGG pathway analysis of common DEGs (**D**, **E**)

The GO functional enrichment analysis of the DEGs with co-morbidities enriched a total of 167 results, and we selected the top 10 results with more significant enrichment of each part to show (Fig. 3 and Supplementary Material S1). KEGG pathway enrichment analysis enriched a total of 17 pathway results that matched the adjusted P of 0.05 (Fig. 3 and Supplementary Material S1).

### PPI network construction and candidate hub gene screening

**Fig. 4:**
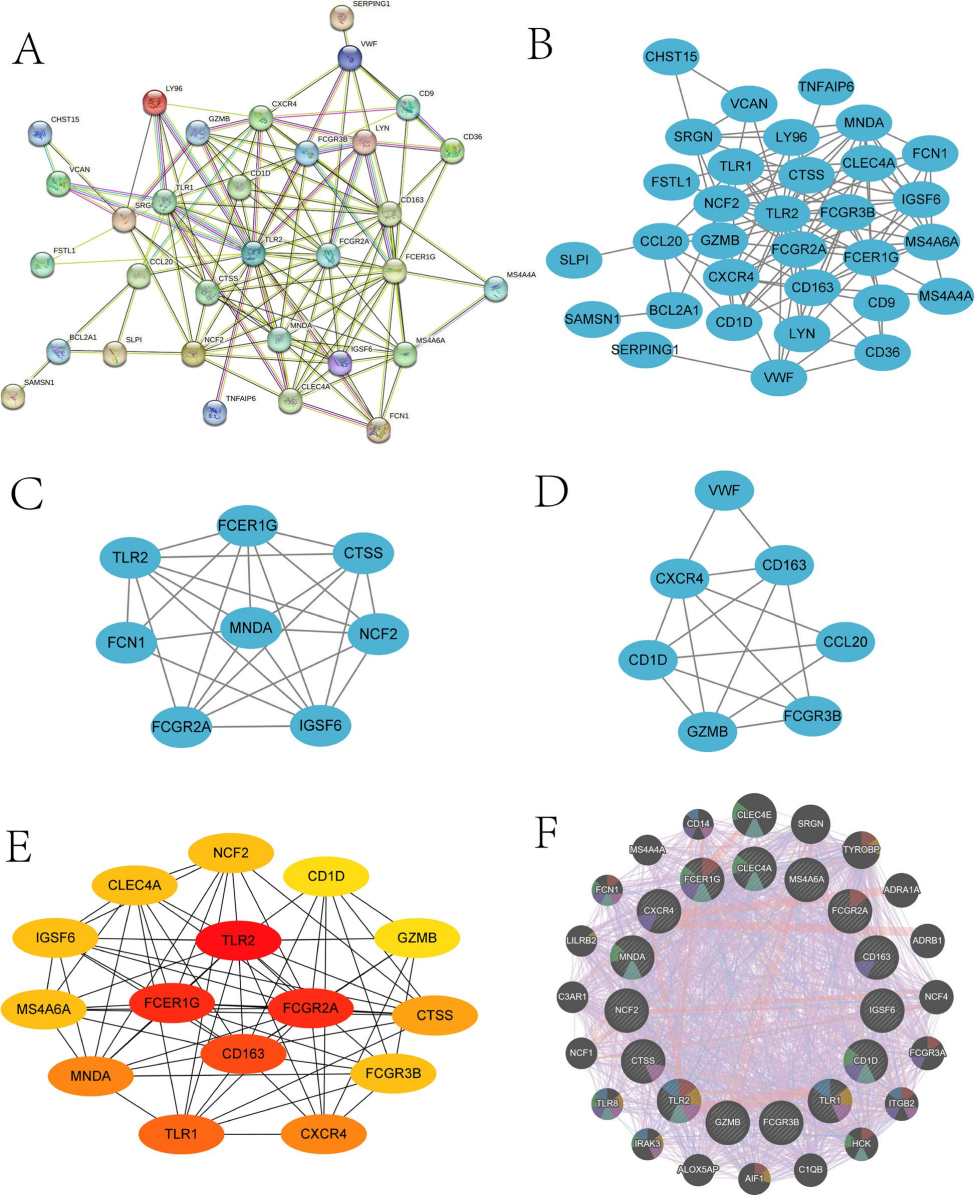
PPI network map and sub-network construction of common DEGs and GeneMANIA co-expression network map of candidate core genes. PPI network map (**A**, **B**); MCODE module sub-network map (**C**–**D**); screening of candidate core genes for cytoHubba plug-in (**E**); GeneMANIA co-expression gene network diagram (**F**)

A PPI network of common DEGs with 27 nodes and 109 edge interaction matches (medium confidence = 0.4; PPI enrichment *p*-value of 1.0e-16) was constructed (Fig. 4A-B), and interestingly, all genes in this network graph were upregulated DEGs. Two interaction subnetworks (Fig. 4C–D) were constructed using the MCODE plugin (degree cutoff = 2, node score cutoff = 0.2, set K-core = 2, and max depth = 100) (subnetwork 1: 8 nodes, 25 edges; subnetwork 2: 7 nodes, 15 edges). Fifteen candidate hub genes were filtered using the Degree module of the cytoHubba plugin (Fig. 4E).

### GO and KEGG analysis of candidate core genes

**Fig. 5:**
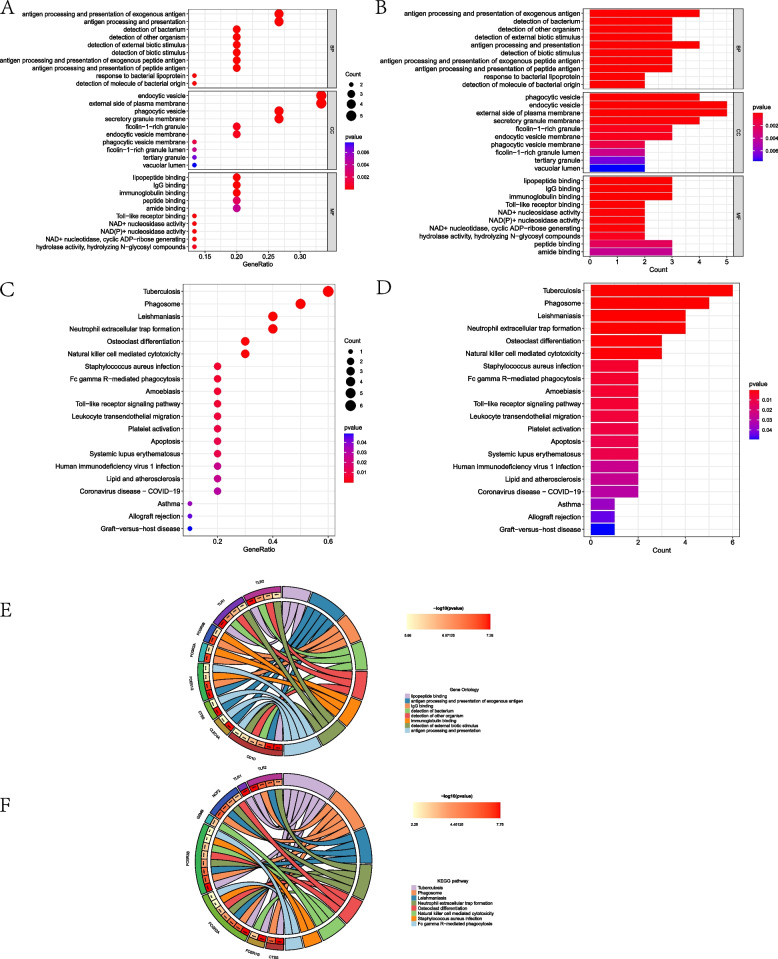
Functional and pathway analysis of candidate core gene enrichment. GO function-enriched bar and bubble plots (**A**, **B**); bar plot and bubble plot of KEGG pathway enrichment (**C**–**D**); GO chord plot (**E**); KEGG chord plot (**F**)

The GO functional enrichment analysis of the DEGs with candidate core genes enriched a total of 86 results, and we selected the top 10 results with more significant enrichment of each part to show. GO functional analysis emphasized the presence of genes mainly in the vesicle membrane and secretory granule membrane involved in antigen processing and presentation, lipopeptide binding, NAD + nucleosidase activity, and Toll-like receptor binding (Fig. 5A, B). KEGG pathway enrichment analysis enriched a total of 14 pathway results that matched the adjusted P of 0.05, and the KEGG pathways analyzed were mainly in the phagosome, neutrophil extracellular trap formation, natural killer cell-mediated cytotoxicity, apoptosis, Fc gamma R-mediated phagocytosis, and Toll-like receptor signaling pathways (Fig. 5C, D). We also used the "circlize" package to plot the chord plot, which can more visually show the interrelationships between genes and gene functions and pathways and share some commonalities, so this chart is ideal for comparing similarities between datasets or different data sets, with nodes distributed around a circle and points connected by arcs to show the relationships, and then assigning weights to each connection (Fig. 5E, F).

### Interaction network of candidate core genes and their co-expression genes

We analyzed the interaction network and associated functions of these candidate core genes on the GeneMANIA website. These genes exhibited a complex interaction network with 11.43% physical interactions, 80.09% co-expression, 0.80% prediction, 6.30% co-localization, 2.87% genetic interactions, and 1.37% pathways. The biological functions of this interaction network are mainly involved in phagocytosis, regulation of interleukin-6 production, pattern recognition receptor signaling pathway, positive regulation of defense response, cell surface, regulation of innate immune response, and toll-like receptor signaling pathway (Fig. 4F and Supplementary Material S2).

### CMap analysis of candidate core genes

**Fig. 6:**
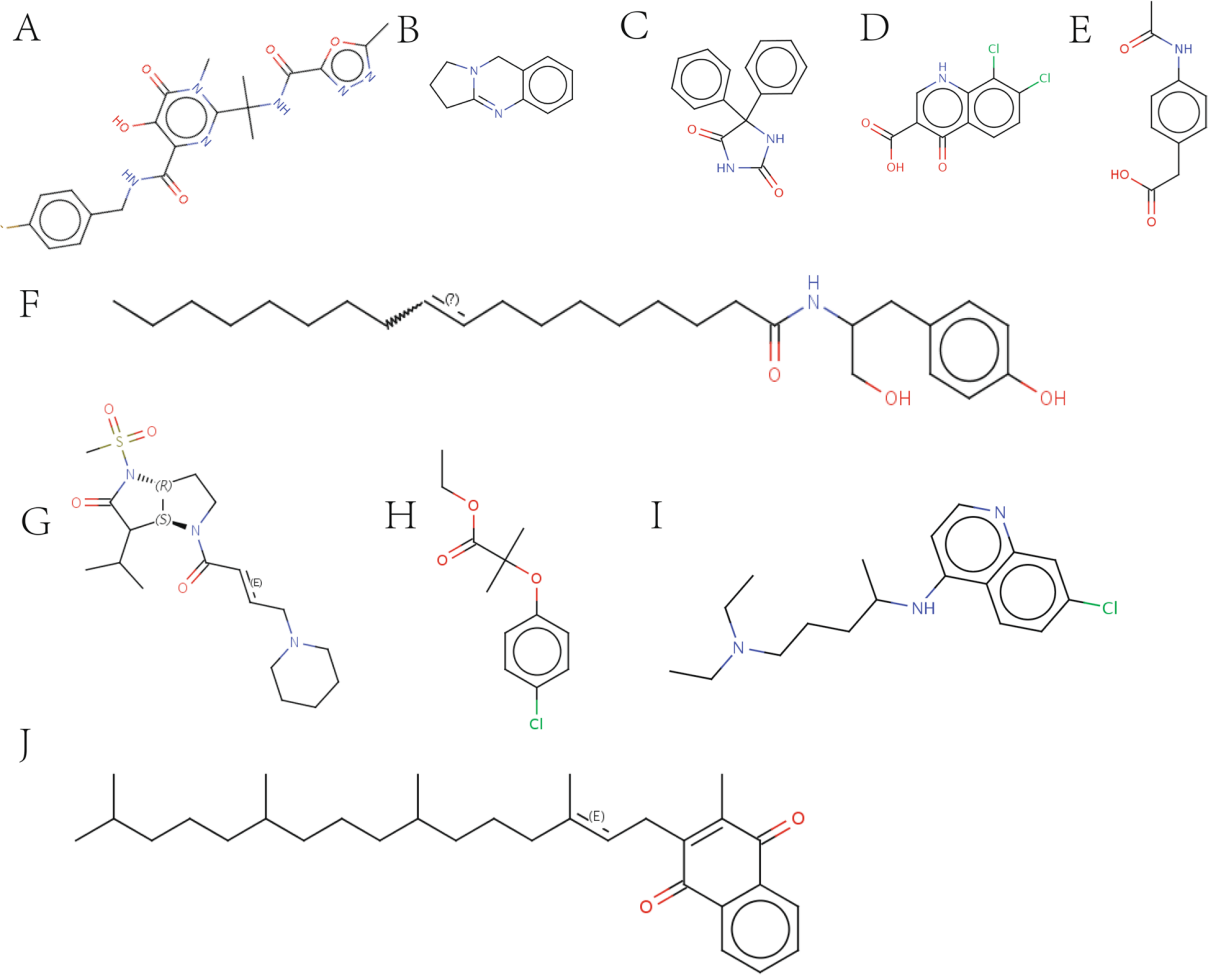
To reveal promising chemical drugs, 15 candidate core genes were submitted to the linkage map (CMap) (https://clue.io/), and the top 10 small molecule compounds with the highest enrichment fraction were identified as potentially promising drugs that could have therapeutic effects on AMI and DN comorbidity. 2D structure diagram of small molecule chemical drugs: **A** raltegravir; **B** Desoxypeganine; **C** Phenytoin; **D** CAY-10577; **E** Actarit; **F** OMDM-2; **G** GW-311616; **H** Clofibrate; **I** Chloroquine; **J** Phylloquinone

We submitted candidate core genes to the CMap database to identify potential small-molecule chemical drugs for AMI and DN co-morbidity core genes. In order to have a more complete understanding of the drug for the disease, we tentatively set the identification score at -80. We selected the top ten small molecule drugs with an identification score close to -100 and well-defined known chemical structures that are more suitable for experimental and clinical applications. The top 10 potential therapeutic compounds (raltegravir, desoxypeganine, phenytoin, CAY-10577, actarit, OMDM-2, GW-311616, clofibrate, chloroquine, and phylloquinone) (Table 1) and the detailed structures of these compounds are shown in Fig. 6.

**Table 1:** Results of CMap

Name	Score	Target	MOA(Description)
raltegravir	-99.93		HIV integrase inhibitor
^Desoxypeganine^	-99.93	ACHE, MAOA	Acetylcholinesterase inhibitor, Monoamine oxidase inhibitor
Phenytoin	-99.93	ABCB1, CYP2B6, CYP2C19, CYP3A4, SCN1A, SCN2A, SCN5A	Hydantoin antiepileptic
CAY-10577	-99.93		Casein kinase inhibitor
actarit	-99.89		Interleukin receptor agonist
OMDM-2	-99.89	GPR119	FAAH inhibitor
GW-311616	-99.89	ELANE	Leukocyte elastase inhibitor
clofibrate	-99.89	PPARA, LPL	PPAR receptor agonist
Chloroquine	-99.89	CYP2C8, GSTA2, MRGPRX1, TLR9, TNF	Antimalarial
Phylloquinone	-99.89	BGLAP, GGCX	Vitamin K, Gamma carboxylase enzyme

### TF Exploration and Certification

**Fig. 7:**
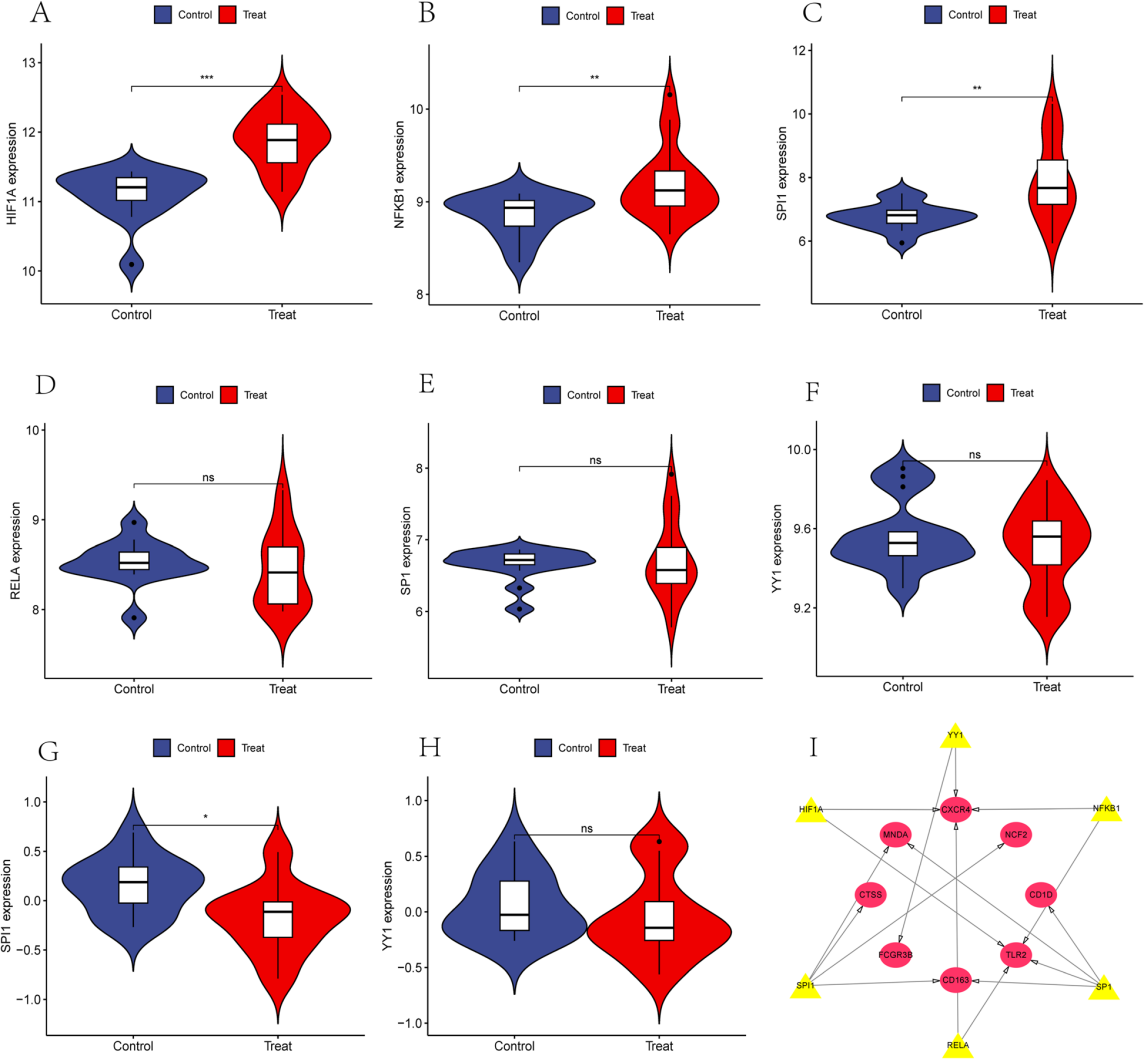
Exploration and validation of TFs and their regulatory networks: **A**–**F** Validation of the six relevant TFs predicted using the TRRUST database is expressed in the GSE66360 training set dataset, with HIF1A, NFKB1, and SPI1 highly expressed in the AMI group. **G**, **H** TFs were expressed in the GSE30529 dataset, and SPI1 was highly expressed in the DN control group. Statistical significance was determined when the *p*-value < 0.05. AMI, acute myocardial infarction; DN, diabetic nephropathy. *p < 0.05; **p < 0.01; *** < 0.001. **I** Regulatory Network of TF: Hub genes are highlighted in red, while TF is highlighted in yellow

We explored the TFs using the TRRUST database (Fig. 7). Thereafter, we validated the expression of these TFs in the training set dataset. Three of the six TFs were highly expressed in GSE66360 (Fig. 7A-F), namely HIF1A, NFKB1, and SPI1. SPI1 was highly expressed in the GSE30529 control (Fig. 7G–H). Subsequently, a total of eight hub genes (TLR2, FCGR3B, CD163, CTSS, MNDA, CXCR4, NCF2, CD1D) and six TFs (SPI1, SP1, HIF1A, YY1, RELA, NFKB1) constructed a network diagram of TFs and hub genes (Fig. 7I).

### Verification of candidate core genes

**Fig. 8:**
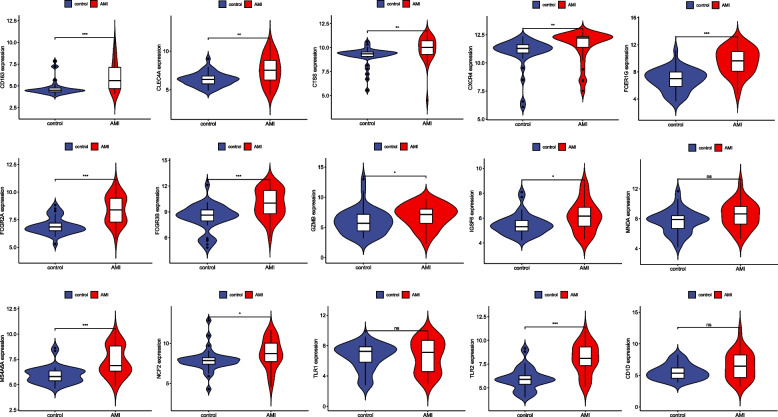
Candidate core gene expression levels in GSE66360 A mean t-test was used to compare the differences in core gene expression between the experimental and control groups. Statistical significance was determined when the *p*-value < 0.05. AMI, acute myocardial infarction; control, healthy control group. *p < 0.05; **p < 0.01; *** < 0.001. ns, *p* > 0.05

**Fig. 9:**
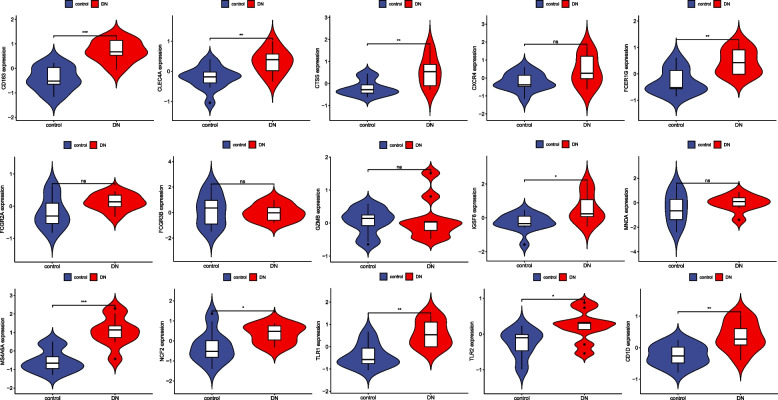
Candidate core gene expression levels in GSE30529. A mean t-test was used to compare the differences in core gene expression between experimental and control groups. Statistical significance was determined when the *p*-value < 0.05. DN, diabetic nephropathy; Control, healthy control group. *p < 0.05; **p < 0.01; *** < 0.001. ns, *p* > 0.05

We validated the expression of candidate core genes in the GSE66360 validation set (AMI) and the GSE30538 dataset (DN). The expression of twelve candidate pivotal genes, TLR2, FCGR2A, FCER1G, CD163, CXCR4, CTSS, CLEC4A, IGSF6, NCF2, FCGR3B, MS4A6A, and GZMB, showed an increasing trend in the GSE6630 validation set (Fig. 8), and the candidate genes TLR2, FCER1G, CD163, TLR1, CTSS, CLEC4A, IGSF6, NCF2, MS4A6A, and CD1D, which were ten genes, showed an increasing trend in the GSE30528 dataset (Fig. 9). Eight AMI and DN co-expressed central genes were finally identified, namely TLR2, FCER1G, CD163, CTSS, CLEC4A, IGSF6, NCF2, and MS4A6A.

### Relationship between co-expressed genes and immune cells

**Fig. 10:**
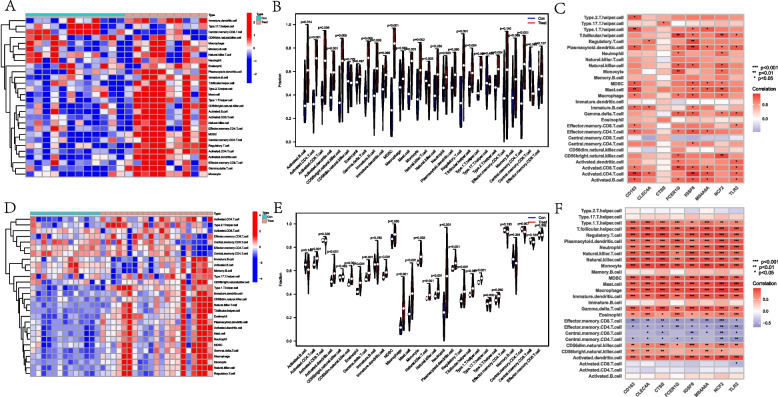
Correlation between DEGs and co-expressed genes with immune cells (**A**-**C**: GSE30529 dataset; **D**-**F**: GSE66360 training set). **A** The correlation between DEGs and immune cells in GSE30529; **B** Differential analysis of immune cells, Activated B cell, Activated CD4 T cell, Activated CD8 T cell, Activated dendritic cell, CD56bright natural killer cell, Gamma delta T cell, Immature B cell, MDSC, Monocyte, NKC, Mast cell, Regulatory T cell, T follicular helper cell, Type 1 T helper cell, Type 2 T helper cell, Central memory CD4 T cells were highly expressed in the experimental group, and central memory CD8 T cells were highly expressed in the control group; **C** The correlation between co-expressed genes and immune cells (GSE30529); **D** The correlation between DEGs and immune cells in GSE66360; **E** Differential analysis of immune cells: activated CD4 T cells, activated dendritic cells, CD56 dim natural killer cell, eosinophils,gamma delta T cell, immature dendritic cell, MDSC, macrophage, mast cell, monocyte, natural killer T cell, natural Neutrophil, Plasmacytoid dendritic cell, Regulatory T cell, T follicular helper cell, and Type 1 T helper cell were highly expressed in the experimental group, and central memory CD4 T cells were highly expressed in the control group; F, the correlation between co-expressed genes and immune cells (GSE66360 training set)

Immune cell content in samples from the AMI training set (GSE66360) and DN training set (GSE30529) was analyzed by gene expression profiling using the GSEABase package and GSVA package. Immunocell heat maps of immune cell expression in the samples were obtained by scoring the immune cell content (Fig. 10A, D). Next, a differential analysis of immune cells was performed to compare the expression of immune cells in the control and test groups (Fig. 10B, E). Finally, we performed immune cell correlation analysis using the Spearman method to explore potential associations between co-expressed genes and immune cells (Fig. 10C, F). Among the eight co-expressed genes and immune correlation analysis in DN, CD163 was associated with Type 2 T helper cells, Type 1 T helper cells, plasmacytoid DC, MDSC, mast cell, macrophage, immature B cell, and activated B. CLEC4A, which is positively correlated with activated CD4 T cells, immature B cells, and regulatory T cells. CTSS was positively correlated with Type 17 T helper cells. FCER1G was positively correlated with activated B cells, activated CD8 T cells, activated dendritic cells, CD56bright NKC, effector memory CD4 T cells, and gamma cells. IGFS6 was positively correlated with activated B cells, activated CD4 T cells, activated CD8 T cells, activated dendritic cells, CD56bright NKC, effector delta T cells, macrophages, monocytes, neutrophils, plasmacytoid DC, T follicular helper cells, Activated CD4 T cells, activated CD8 T cells, central memory CD4 T cells, effector memory CD4 T cells, gamma delta T cells, immature B cells, MDSC, mast cell macrophages, plasmacytoid DC, regulatory T cells, T follicular helper cells, and Type 1 T helper cells. MS4A6A is positively associated with activated B cells, activated CD4 T cells, activated CD8 T cells, effector memory CD4 T cells, MDSC, mast cells, NKC, plasmacytoid DC, and Type 1 T helper cells. NCF2 is positively correlated with CD56-bright natural killer cells, gamma delta T cells, MDSC, NKC, plasmacytoid DC, and Type 1 T helper cells. TLR2 was positively correlated with activated B cells, activated CD4 T cells, activated CD8 T cells, activated dendritic cells, effector memory CD8 T cells, gamma delta T cells, plasmacytoid dendritic cells, and T follicular helper cells. In the immune correlation analysis of AMI, eight co-expressed genes were positively correlated with CD56 dim NKC, CD56 bright NKC, activated DC, eosinophils, gamma delta T cells, and immature dendritic cells, macrophages, mast cells, MDSC, monocytes, NKC, natural killer T cells, neutrophils, plasmacytoid dendritic cells, regulatory T cells, T follicular helper cells, and Type 1 T helper cells; negatively correlated with central memory CD4 T cell, effector memory CD4 T cell, and central and effector memory CD8 T cells.

## Discussion

CHD is the most common cause of death worldwide, causing 8.9 million deaths in 2019 alone [19]. AMI is a potentially fatal presentation of CHD and is a main component of CHD deaths [20], and its complications can cause a serious economic and social burden. DN is essentially a chronic microvascular complication of diabetes and a major cause of ESRD, the overall prevalence of DN in China is 8.95% [21]. AMI and DN co-morbidities are very commonly seen clinically and have a poor prognosis, and bioinformatics studies of their co-morbidities have not been reported. Our study provides some contributions to the identification of novel biomarkers of susceptibility to diabetic nephropathy and AMI co-morbidity and useful potential therapeutic targets. Transcriptome difference analysis relies on three main R packages: DESeq2, edgeR, and limma. These packets extract large differences in DGEs, thus enhancing the identification of DEGs using intersection analysis methods. However, DESeq2 and edgeR are suitable for analyzing small data samples, and the false positive rate increases significantly with large samples. In this study, we used the most commonly used limma package for analysis to improve the accuracy of DEGs and enrichment analysis.

In this study, we report the following bioinformatics studies on co-morbidities: We identified and validated eight co-morbid genes, namely TLR2, FCER1G, CD163, CTSS, CLEC4A, IGSF6, NCF2, and MS4A6A. TLR2 is a gene encoding a Toll-like receptor family of proteins, and intervention in the TLR2 signaling pathway may be beneficial in the treatment of DN [22]. TLR2 activates inflammatory responses by activating the TLR2-Myd88-NF-κB pathway, also activates immune cells to promote apoptosis [23], and is associated with the severity of coronary artery disease, plaque instability, and myocardial infarction in myocardial injury [24]. High expression of FCER1G in the plasma of patients with plaque rupture [25] may be associated with atheromatous plaque instability by unknown mechanisms. CD163 is expressed only in monocytes and macrophages, and levels of the soluble form of CD163 (sCD163) are elevated in low-grade inflammation [26], which may be involved in chronic inflammatory responses and possibly in AMI and DN development through the induction of foam cell formation and local inflammatory responses. CTSS degrades damaged or unwanted proteins in lysosomes, regulates antigen presentation in atherosclerosis [27], and mediates phagosome involvement in atherosclerosis progression through macrophages [28]. CTSS is also a biomarker of chronic kidney disease, and as the glomerular filtration rate decreases, CTSS increases, exacerbating inflammation-associated endothelial dysfunction [29]. CTSS is closely associated with diabetes and complications (cardiovascular disease, retinopathy, diabetic nephropathy, and other microangiopathies) [30], impairs endothelial function through PAR2 activation, and promotes podocyte reduction [31]. CLEC4A encodes C-type lectin, which is involved in cell adhesion, intercellular signaling, glycoprotein renewal, and inflammatory and immune responses (https://www.ncbi.nlm.nih.gov/gene/). IGSF6, immunoglobulin superfamily member 6, may be a plasma membrane component involved in cross-modal signaling. NCF2 may be involved in iron death in cells from DN patients [32]. MS4A6A is recognized to be associated with aging and neurodegenerative diseases, and bioinformatic analysis has revealed its high expression in DN, but the exact mechanism is unknown.

GO function is enriched in the vesicle membrane and secretory membrane and is involved in antigen processing and presentation, lipopeptide binding, NAD+ nucleosidase activity, and Toll-like receptor binding. Atherosclerosis is a chronic inflammatory vascular disease [33]; both AMI and DN are essentially atherosclerotic diseases; and most immune cells are involved in the atherosclerotic process [34]. Macrophages are considered the main immune cell type in atherosclerotic plaques [35], acting as antigen-presenting cells and presenting antigens to naive CD8 + T cells and naive CD4 + T cells [36]. CD8 + killer T cells induce apoptosis and necrosis of target cells, exacerbating progression in atherosclerotic plaques [37], while CD4T cell subsets can influence atherosclerotic progression through immune activation or immunosuppression [38]. Macrophages can also be involved in atherosclerotic plaque formation through toll-like receptor activation by oxLDL [39]. Toll-like receptor 4 (TLR4), a cellular inflammatory factor, can induce the expression and release of acquired immune-induced inflammatory factors by initiating natural immunity and stimulating the production of cytokines and chemokines through the corresponding signaling pathways. release, which in turn induces DN and atherogenesis [40]. Macrophage infiltration around renal tubular epithelial cells is considered a hallmark of DN, and DN or lipotoxicity promotes the increased release of extracellular vesicles, which were found to induce apoptosis of injured renal tubular epithelial cells through a death receptor 5 (DR5)-dependent process [41]. Nicotinamide adenine dinucleotide (NAD+) is a carrier of high-energy electrons generated by oxidative metabolism in vivo and serves as a cofactor for most oxidative enzymes with redox and signaling functions. NAD+ dynamic balance has a key role in cellular catabolism and anabolism and is also an important cofactor for redox reactions in the cytoplasm and mitochondria, regulating a variety of cellular processes, including the cellular stress response. It is also an important cofactor in the cytoplasm and mitochondrial redox reactions, regulating various cellular processes including cellular stress response, mitochondrial homeostasis, and calcium signaling. The kidney is second only to the heart in the number of mitochondria and has a high oxidative metabolism and a high demand for NAD+ . NAD+ is a determinant of aging [42], and alterations in NAD+ levels have been detected in diabetic nephropathy in studies suggesting a possible involvement in the development of diabetic nephropathy, but no assessment of NAD+ distribution in the kidney is available [43]. Studies have shown that NAD+ supplementation reduces myocardial infarction size during cardiac ischemia through the longevity protein Sirt1. This process, moreover, relies on the longevity protein Sirt1 to act [44]. A follow-up study found that NAD + supplementation with schizomycin inhibited longevity protein activity but still reduced myocardial infarction in mice, suggesting that the cardioprotective effect of NAD+ supplementation is not only dependent on longevity protein. Further studies confirmed [45] that NAD+ supplementation activates intracellular glycolysis and thus generates large amounts of energy, which acts to protect the myocardium and reduce myocardial infarction through glycolysis in the presence of sufficient glucose in the body.

KEGG pathway enrichment focuses on the phagosome, neutrophil extracellular trap formation, natural killer cell-mediated cytotoxicity, apoptosis, Fc gamma R-mediated phagocytosis, and Toll-like receptor signaling pathways. Pathway enrichment analysis suggests that FcγR-mediated phagocytosis and Toll-like receptor signaling pathways are involved in the pathogenesis of AMI and DN. FcγR, a receptor for the Fc portion of IgG, activates macrophages by mediating the activation of the mitogen-activated protein kinase signaling pathway by low-density lipoprotein immune complexes (LDL-ICs) [46]. Macrophage polarization toward the M1 phenotype increases the risk of AMI pathogenesis and rupture and also the formation of atherosclerotic plaques by taking up LDL-ICs and converting them into foam cells [47]. FcγR activates and promotes phagocytosis of macrophages, induces foam cell aggregation, and promotes the development of atherosclerotic plaques [23], inducing and exacerbating AMI and DN. Neutrophil The formation of extracellular traps (NETs) in granulocytes can be triggered by various stimuli induced by contact with surface receptors (cytokine receptors, Fcγ receptors, toll-like receptors, complement receptors, etc.) and adenosine receptors, causing an increase in intracellular Ca2 + concentration, which activates protein kinase c (PKC), leading to reactive oxygen species (ROS) through NADPH oxidase and/or mitochondrial activation production, resulting in organismal damage [48].

Meanwhile, we screened small molecule drugs for co-morbidities through the CMap (https://clue.io/) website and selected the top 10 small molecule drugs, namely raltegravir, desoxypeganine, phenytoin, CAY-10577, actarit, OMDM-2, GW-311616, clofibrate, chloroquine, and phylloquinone, which may become new approaches for the treatment of AMI and DN co-morbidities. As the first integrase inhibitor approved for the treatment of HIV infection, Raltegravir is gradually being discovered to have effects other than anti-HIV. Studies have shown that Raltegravir may be effective in treating multiple myeloma by promoting DNA damage-induced apoptosis [49]. Another study showed that Raltegravir was effective in treating human T-cell lymphoblastic virus-1 (HTLV-1)-associated myelopathy/tropical spastic paraplegia (HAM/TSP). Although it did not significantly reduce the high proviral load, it produced therapeutic effects on this chronic progressive disease by reducing the expression of CD4+ CD25+ T cells, antibody-secreting B cells, activated CD8+ T cells, and IFN-γ [50]. We speculate that Raltegravir may play an anti-arterial inflammatory role by regulating inflammatory cells and inflammatory factors, but no relevant reports have been reported to date. Desoxypeganine (DOP) is a natural alkaloid. It is not only a cholinesterase inhibitor but also a selective inhibitor of monoamine oxidase A. Oral DOP is well tolerated and safe for smoking and alcohol cessation [51]. Cholinesterase plays a key role in lipid metabolism and is also a predisposing factor for metabolic diseases [52]. Cholinesterase activity is usually higher in diabetic patients than in hyperlipidemia patients [53]. Galantamine, a cholinesterase inhibitor, can reduce inflammation and insulin resistance in metabolic syndrome and improve diabetic kidney damage and renal function [54]. The cholinesterase inhibitor pyridostigmine can enhance cardiac parasympathetic tone and induce an anti-inflammatory response in the heart, thereby promoting cardiac recovery after AMI [55]. High-density lipoprotein (HDL) cholesterol is negatively associated with coronary heart disease, and phenytoin, a drug that can raise HDL levels [56], may be related to HDL's involvement in atherosclerosis mechanisms. Actarit is a drug for the treatment of rheumatoid arthritis, and its main target of action is carbonic anhydrase II [57]. This target is very important in the kidney and osteoclasts, and its main function is to maintain acid-base balance in the body as well as normal bone remodeling [58]. At the same time, Actarit's research in the treatment of heart disease and other diseases is also being repositioned [57]. OMDM-2 is a cannabinoid transporter inhibitor. Angiotensin II releases endocannabinoids by activating the AT1 receptor [59]. The mechanism of the cardiovascular system's response to endocannabinoids is complex, and there are still many unsolved mysteries. It has been suggested that cannabinoid-1 receptor (CB1R) activation is associated with oxidative low-density lipoprotein formation and inflammatory response [60], while CB2R agonists attenuate the inflammatory response of human coronary endothelial cells caused by CB1R activation [61]. Overactivation of CB1R in the renal endocannabinoid system contributes to DN development. Moreover, CB1R blockers have been shown to improve renal insufficiency in mouse models of diabetes [62]. In podocytes, CB1R deletion alleviates glomerular and tubular dysfunction in mouse models of diabetic nephropathy [63]. Animal experiments have shown that neutrophil elastase (NE) is involved in the formation of foam cells and the progression of atherosclerosis, and the specific neutrophil elastase inhibitor GW-311616 significantly reduces atherosclerosis [64]. Clofibrate is a peroxisome proliferator-activated receptor-alpha (PPARα) agonist that reduces the inflammatory response and modulates the renin-angiotensin system. One week of Clofibrate treatment reduced angiotensin II and the angiotensin-converting enzyme, AT1, while bradykinin, ACE-2, and AT2 receptors were elevated, nitric oxide levels increased, and fibrosis and structural damage caused by myocardial infarction improved [65]. Clofibrate also reduced glomerular transforming growth factor (TGF)-β1-induced increased albumin permeability in DN rats [66] or may be used for early treatment of DN. Chloroquine (CQ) is an autophagy inhibitor that inhibits lipid deposition-related kidney injury mediated by autophagy deficiency in DN [67]. Studies have shown that hydroxychloroquine has a role in reducing the risk of cardiovascular disease events in patients with traditional risk factors [68], while chloroquine diphosphate promotes caspase-3 and caspase-9 activity and increases AMI cardiomyocyte apoptosis [69]. Vitamin K is an essential bioactive substance required by the body. According to its main structure, it is divided into phylloquinones (K1) and menaquinones (K2). Phylloquinone is mainly derived from leafy green vegetables and can be converted into menaquinone-4 (MK4) in vivo. It can effectively inhibit lipid peroxidation and its mediated iron death. It can effectively prevent tissue damage in the model of hepatic and renal ischemia–reperfusion injury. It may be one of the oldest natural iron-resistant quinones [70]. K2 is potent in preventing arterial calcification, reducing the risk of diabetes, and improving kidney function [71]. In general, desoxypeganine, actarit, OMDM-2, GW-311616, clofibrate, and phylloquinones may play a greater role in the treatment of DN and coronary heart disease.

We also constructed a network map consisting of eight hub genes (TLR2, FCGR3B, CD163, CTSS, MNDA, CXCR4, NCF2, and CD1D) and six transcription factors (SPI1, SP1, HIF1A, YY1, RELA, and NFKB1) using the TRRUST dataset and examined the TF expression in the training set. A total of three AMI-related transcription factors (HIF1A, NFKB1, and SPI1) and one DN-related TF (SPI1) were identified, suggesting that SPI1 may play opposite roles in the two diseases, a conclusion that needs to be further confirmed in external data. To validate transcription factor expression, we initially constructed myocardial infarction models and diabetic nephropathy models to confirm whether transcription factor expression in the models was consistent with bioinformatics predictions. The procedure begins with bioinformatics analysis that screens for conserved transcription factor sequences, transcription factor core regions, signal peptides, protein glycosylation checkpoints, phosphorylation checkpoints, protein kinase binding checkpoints, and protein affinity/hydrophobicity. Transcription factors are thoroughly examined for their overall transcriptional activation using a dual luciferase detection system. Gene expression is further verified by overexpressing or silencing transcription factors, and the regulatory relationship between transcription factors and target genes is elucidated in relation to phenotype. Protein-DNA interactions are used to identify the DNA sequence to which the transcription factor binds, and chromatin immunoprecipitation (TF-CHIP) is used to determine whether the transcription factor binds to gene promoter sequences. This process will be further refined and completed in future work.

Our findings contribute to the clarification of the mechanisms underlying the association between AMI and DN. However, the study still has some shortcomings. Our findings need further confirmation with external data; core genes need further experimental validation in vitro models; and some of the genes enriched are not yet reported in the literature on AMI or DN. Supplementing these shortcomings will be the focus of our future studies.

## Conclusion

We identified and validated eight co-expressed central genes and three TFs of AMI and DN. The preliminary screening of small-molecule chemotherapeutics for co-morbidities provided new directions for co-morbidity understanding and research and new ideas for treatment. TLR2, FCER1G, CD163, CTSS, CLEC4A, IGSF6, NCF2, and MS4A6A are eight co-expressed central genes that were enriched in an analysis focusing on NAD+ nucleosidase activity and Toll-like receptor binding in the phagosome. neutrophil extracellular trap formation, natural killer cell-mediated cytotoxicity, apoptosis, Fc gamma R-mediated phagocytosis, and Toll-like receptor signaling pathways.

### Supplementary Information

Supplementary Material 1.

Supplementary Material 2.

## Data Availability

The current dataset analyzed in the study can be found in the GEO database. GSE66360: https://www.ncbi.nlm.nih.gov/geo/query/acc.cgi?acc=GSE66360. GSE30528: https://www.ncbi.nlm.nih.gov/geo/query/acc.cgi?acc=GSE30528. GSE30529: https://www.ncbi.nlm.nih.gov/geo/query/acc.cgi?acc=GSE30529.
