# Supplementary material for: Identification of co-expressed central genes and transcription factors in acute myocardial infarction and diabetic nephropathy
Source: BMC Med Genomics. 2024 May 20;17:134. doi: 10.1186/s12920-024-01906-7 (PMC11103847; doi:10.1186/s12920-024-01906-7)
Supplement: Supplementary file 1 — Supplementary Material 1. [file 12920_2024_1906_MOESM1_ESM.pdf]

GO enrichment analysis results for differentially expressed genes

ONTOLOGY	ID	Description	p.adjust	qvalue	geneID	Count
BP	GO:0002221	pattern recognition receptor signaling pathway	7.70E-05	5.38E-05	CTSS/LYN/FCN1/LY96/TLR2/TLR1/CD36	7
BP	GO:0098581	detection of external biotic stimulus	7.70E-05	5.38E-05	LY96/TLR2/TLR1/CD1D	4
BP	GO:0002224	toll-like receptor signaling pathway	7.70E-05	5.38E-05	CTSS/LYN/LY96/TLR2/TLR1/CD36	6
BP	GO:0009595	detection of biotic stimulus	0.000307467	0.000214863	LY96/TLR2/TLR1/CD1D	4
BP	GO:0032493	response to bacterial lipoprotein	0.000307467	0.000214863	TLR2/TLR1/CD36	3
BP	GO:0032490	detection of molecule of bacterial origin	0.000341129	0.000238386	LY96/TLR2/TLR1	3
BP	GO:0019884	antigen processing and presentation of exogenous antigen	0.000521581	0.000364488	CTSS/CLEC4A/FCER1G/CD1D	4
BP	GO:0016045	detection of bacterium	0.000697791	0.000487626	TLR2/TLR1/CD1D	3
BP	GO:0034134	toll-like receptor 2 signaling pathway	0.000697791	0.000487626	LYN/TLR2/TLR1	3
BP	GO:0007596	blood coagulation	0.000698811	0.000488339	LYN/SERPING1/VWF/FCER1G/CD9/CD36	6
BP	GO:0050817	coagulation	0.000698811	0.000488339	LYN/SERPING1/VWF/FCER1G/CD9/CD36	6
BP	GO:0007599	hemostasis	0.000698811	0.000488339	LYN/SERPING1/VWF/FCER1G/CD9/CD36	6
BP	GO:0031663	lipopolysaccharide-mediated signaling pathway	0.000698811	0.000488339	LYN/LY96/TLR2/CD36	4
BP	GO:0002831	regulation of response to biotic stimulus	0.000698811	0.000488339	MNDA/LYN/FCN1/LY96/SERPING1/CD1D/CD36	7
BP	GO:0098543	detection of other organism	0.000698811	0.000488339	TLR2/TLR1/CD1D	3
BP	GO:0071404	cellular response to low-density lipoprotein particle stimulus	0.000882413	0.000616642	FCER1G/CD9/CD36	3
BP	GO:0034121	regulation of toll-like receptor signaling pathway	0.001314474	0.000918573	LYN/TLR2/TLR1/CD36	4
BP	GO:0042060	wound healing	0.002052249	0.00143414	LYN/SERPING1/CXCR4/VWF/FCER1G/CD9/CD36	7

BP	GO:0032640	tumor necrosis factor production	0.002052249	0.00143414	CLEC4A/LY96/TLR2/TLR1/CD36	5
BP	GO:0032680	regulation of tumor necrosis factor production	0.002052249	0.00143414	CLEC4A/LY96/TLR2/TLR1/CD36	5
BP	GO:0002833	positive regulation of response to biotic stimulus	0.002052249	0.00143414	MNDA/LYN/FCN1/LY96/CD1D	5
BP	GO:0071706	tumor necrosis factor superfamily cytokine production	0.002052249	0.00143414	CLEC4A/LY96/TLR2/TLR1/CD36	5
BP	GO:1903555	regulation of tumor necrosis factor superfamily cytokine production	0.002052249	0.00143414	CLEC4A/LY96/TLR2/TLR1/CD36	5
BP	GO:0032103	positive regulation of response to external stimulus	0.002052249	0.00143414	MNDA/LYN/FCN1/LY96/CXCR4/TLR2/CD1D	7
BP	GO:0006909	phagocytosis	0.002052249	0.00143414	LYN/FCN1/TLR2/NCF2/FCER1G/CD36	6
BP	GO:0055094	response to lipoprotein particle	0.002073205	0.001448785	FCER1G/CD9/CD36	3
BP	GO:0050869	negative regulation of B cell activation	0.002339115	0.001634606	MNDA/LYN/SAMSN1	3
BP	GO:0001819	positive regulation of cytokine production	0.002339115	0.001634606	MNDA/FCN1/LY96/TLR2/TLR1/FCER1G/CD36	7
BP	GO:0071402	cellular response to lipoprotein particle stimulus	0.002339115	0.001634606	FCER1G/CD9/CD36	3
BP	GO:0050764	regulation of phagocytosis	0.002339115	0.001634606	FCN1/TLR2/FCER1G/CD36	4
BP	GO:0032760	positive regulation of tumor necrosis factor production	0.002440004	0.001705109	LY96/TLR2/TLR1/CD36	4
BP	GO:0062207	regulation of pattern recognition receptor signaling pathway	0.002520528	0.00176138	LYN/TLR2/TLR1/CD36	4
BP	GO:0019882	antigen processing and presentation	0.002520528	0.00176138	CTSS/CLEC4A/FCER1G/CD1D	4
BP	GO:1903557	positive regulation of tumor necrosis factor superfamily cytokine production	0.002520528	0.00176138	LY96/TLR2/TLR1/CD36	4
BP	GO:0002478	antigen processing and presentation of exogenous peptide antigen	0.002520528	0.00176138	CTSS/CLEC4A/FCER1G	3

BP	GO:0002237	response to molecule of bacterial origin	0.003032711	0.002119301	LYN/LY96/TLR2/TLR1/SLPI/CD36	6
BP	GO:0008037	cell recognition	0.00344603	0.002408134	VCAN/FCN1/CXCR4/CD9/CD36	5
BP	GO:0071219	cellular response to molecule of bacterial origin	0.003637658	0.002542047	LYN/LY96/TLR2/TLR1/CD36	5
BP	GO:0030595	leukocyte chemotaxis	0.003890171	0.002718506	LYN/TNFAIP6/CXCR4/FCER1G/CCL20	5
BP	GO:0045088	regulation of innate immune response	0.003890171	0.002718506	MNDA/LYN/FCN1/SERPING1/CD1D	5
BP	GO:0097529	myeloid leukocyte migration	0.003945261	0.002757004	LYN/TNFAIP6/FCER1G/CD9/CCL20	5
BP	GO:0050878	regulation of body fluid levels	0.004024095	0.002812094	LYN/SERPING1/VWF/FCER1G/CD9/CD36	6
BP	GO:0050900	leukocyte migration	0.004215004	0.002945504	LYN/TNFAIP6/CXCR4/FCER1G/CD9/CCL20	6
BP	GO:0030168	platelet activation	0.004215004	0.002945504	LYN/VWF/FCER1G/CD9	4
BP	GO:0006968	cellular defense response	0.004215004	0.002945504	MNDA/LY96/NCF2	3
BP	GO:0010543	regulation of platelet activation	0.004215004	0.002945504	LYN/FCER1G/CD9	3
BP	GO:0070391	response to lipoteichoic acid	0.00431969	0.00301866	TLR2/CD36	2
BP	GO:0071223	cellular response to lipoteichoic acid	0.00431969	0.00301866	TLR2/CD36	2
BP	GO:0061041	regulation of wound healing	0.00431969	0.00301866	SERPING1/CXCR4/CD9/CD36	4
BP	GO:0071216	cellular response to biotic stimulus	0.004598294	0.003213352	LYN/LY96/TLR2/TLR1/CD36	5
BP	GO:0045089	positive regulation of innate immune response	0.005157657	0.003604243	MNDA/LYN/FCN1/CD1D	4
BP	GO:0001867	complement activation, lectin pathway	0.005761519	0.00402623	FCN1/SERPING1	2
BP	GO:0034135	regulation of toll-like receptor 2 signaling pathway	0.005761519	0.00402623	LYN/TLR1	2
BP	GO:0032757	positive regulation of interleukin-8 production	0.006348673	0.004436542	FCN1/TLR2/TLR1	3
BP	GO:0002218	activation of innate immune response	0.006413615	0.004481924	MNDA/LYN/FCN1	3
BP	GO:0048002	antigen processing and presentation of peptide antigen	0.006413615	0.004481924	CTSS/CLEC4A/FCER1G	3

BP	GO:0014015	positive regulation of gliogenesis	0.007866065	0.005496917	LYN/CXCR4/TLR2	3
BP	GO:0030193	regulation of blood coagulation	0.008064769	0.005635774	SERPING1/CD9/CD36	3
BP	GO:0031349	positive regulation of defense response	0.00819577	0.00572732	MNDA/LYN/FCN1/TLR2/CD1D	5
BP	GO:1903034	regulation of response to wounding	0.00819577	0.00572732	SERPING1/CXCR4/CD9/CD36	4
BP	GO:0042063	gliogenesis	0.00819577	0.00572732	LYN/CXCR4/TLR2/CD9/LAMB1	5
BP	GO:1900046	regulation of hemostasis	0.00819577	0.00572732	SERPING1/CD9/CD36	3
BP	GO:0050766	positive regulation of phagocytosis	0.008741137	0.00610843	FCN1/FCER1G/CD36	3
BP	GO:0050818	regulation of coagulation	0.008883324	0.006207792	SERPING1/CD9/CD36	3
BP	GO:0060326	cell chemotaxis	0.008883324	0.006207792	LYN/TNFAIP6/CXCR4/FCER1G/CCL20	5
BP	GO:0032496	response to lipopolysaccharide	0.01151846	0.008049262	LYN/LY96/TLR2/SLPI/CD36	5
BP	GO:0030889	negative regulation of B cell proliferation	0.011698217	0.008174879	MNDA/LYN	2
BP	GO:0070098	chemokine-mediated signaling pathway	0.014337187	0.010019028	LYN/CXCR4/CCL20	3
BP	GO:0032288	myelin assembly	0.015283325	0.010680203	TLR2/CD9	2
BP	GO:1990868	response to chemokine	0.016725967	0.011688341	LYN/CXCR4/CCL20	3
BP	GO:1990869	cellular response to chemokine	0.016725967	0.011688341	LYN/CXCR4/CCL20	3
BP	GO:0002697	regulation of immune effector process	0.016725967	0.011688341	LYN/FCN1/SERPING1/CD1D/CD36	5
BP	GO:0002755	MyD88-dependent toll-like receptor signaling pathway	0.016725967	0.011688341	TLR2/TLR1	2
BP	GO:0038095	Fc-epsilon receptor signaling pathway	0.016725967	0.011688341	LYN/FCER1G	2
BP	GO:0050866	negative regulation of cell activation	0.016725967	0.011688341	MNDA/LYN/CD9/SAMSN1	4
BP	GO:0032755	positive regulation of interleukin-6 production	0.016725967	0.011688341	TLR2/TLR1/CD36	3
BP	GO:0042742	defense response to bacterium	0.016725967	0.011688341	TLR2/FCER1G/SLPI/CD36/CCL20	5
BP	GO:0071222	cellular response to lipopolysaccharide	0.016725967	0.011688341	LYN/LY96/TLR2/CD36	4
BP	GO:0048709	oligodendrocyte differentiation	0.016739578	0.011697853	LYN/CXCR4/TLR2	3
BP	GO:0048714	positive regulation of	0.017050751	0.011915305	CXCR4/TLR2	2

		oligodendrocyte differentiation				
BP	GO:0032677	regulation of interleukin-8 production	0.017777906	0.012423451	FCN1/TLR2/TLR1	3
BP	GO:0032637	interleukin-8 production	0.018056324	0.012618013	FCN1/TLR2/TLR1	3
BP	GO:0002685	regulation of leukocyte migration	0.018195982	0.012715609	LYN/TNFAIP6/CD9/CCL20	4
BP	GO:0002253	activation of immune response	0.018307627	0.012793627	MNDA/LYN/FCN1/SERPING1/FCER1G	5
BP	GO:0030593	neutrophil chemotaxis	0.01839981	0.012858046	TNFAIP6/FCER1G/CCL20	3
BP	GO:0014013	regulation of gliogenesis	0.018683144	0.013056044	LYN/CXCR4/TLR2	3
BP	GO:0002274	myeloid leukocyte activation	0.019072847	0.013328374	LYN/TLR2/TLR1/FCER1G	4
BP	GO:0031664	regulation of lipopolysaccharide-mediated signaling pathway	0.019452675	0.013593803	LY96/CD36	2
BP	GO:0034123	positive regulation of toll-like receptor signaling pathway	0.020631258	0.014417414	TLR2/TLR1	2
BP	GO:0002474	antigen processing and presentation of peptide antigen via MHC class I	0.021355545	0.014923556	CLEC4A/FCER1G	2
BP	GO:0019886	antigen processing and presentation of exogenous peptide antigen via MHC class II	0.021355545	0.014923556	CTSS/FCER1G	2
BP	GO:0090330	regulation of platelet aggregation	0.021355545	0.014923556	LYN/CD9	2
BP	GO:0002220	innate immune response activating cell surface receptor signaling pathway	0.022552717	0.015760156	LYN/FCN1	2
BP	GO:0002758	innate immune response-activating signal transduction	0.023768533	0.016609786	LYN/FCN1	2
BP	GO:0031623	receptor internalization	0.024042711	0.016801385	FCER1G/CD9/CD36	3
BP	GO:0002699	positive regulation of immune effector process	0.02423685	0.016937052	LYN/FCN1/CD1D/CD36	4
BP	GO:0002431	Fc receptor mediated stimulatory signaling pathway	0.02423685	0.016937052	LYN/FCER1G	2
BP	GO:0038094	Fc-gamma receptor signaling pathway	0.02423685	0.016937052	LYN/FCER1G	2

BP	GO:0002495	antigen processing and presentation of peptide antigen via MHC class II	0.025457506	0.017790063	CTSS/FCER1G	2
BP	GO:0002683	negative regulation of immune system process	0.026341075	0.018407514	MNDA/LYN/TNFAIP6/SERPING1/SAMSN1	5
BP	GO:0071621	granulocyte chemotaxis	0.027141593	0.018966927	TNFAIP6/FCER1G/CCL20	3
BP	GO:1990266	neutrophil migration	0.027141593	0.018966927	TNFAIP6/FCER1G/CCL20	3
BP	GO:0002504	antigen processing and presentation of peptide or polysaccharide antigen via MHC class II	0.027141593	0.018966927	CTSS/FCER1G	2
BP	GO:0071711	basement membrane organization	0.027141593	0.018966927	CTSS/LAMB1	2
BP	GO:0034110	regulation of homotypic cell-cell adhesion	0.028373951	0.019828116	LYN/CD9	2
BP	GO:0002443	leukocyte mediated immunity	0.028373951	0.019828116	LYN/SERPING1/FCER1G/CD1D/GZMB	5
BP	GO:0042552	myelination	0.033585024	0.023469688	CXCR4/TLR2/CD9	3
BP	GO:0007272	ensheathment of neurons	0.034291669	0.023963501	CXCR4/TLR2/CD9	3
BP	GO:0008366	axon ensheathment	0.034291669	0.023963501	CXCR4/TLR2/CD9	3
BP	GO:0032735	positive regulation of interleukin-12 production	0.034477035	0.024093038	TLR2/CD36	2
BP	GO:0034142	toll-like receptor 4 signaling pathway	0.034477035	0.024093038	LYN/LY96	2
BP	GO:0014003	oligodendrocyte development	0.035160349	0.024570547	LYN/TLR2	2
BP	GO:0045687	positive regulation of glial cell differentiation	0.035160349	0.024570547	CXCR4/TLR2	2
BP	GO:0048713	regulation of oligodendrocyte differentiation	0.035160349	0.024570547	CXCR4/TLR2	2
BP	GO:0062208	positive regulation of pattern recognition receptor signaling pathway	0.038110877	0.026632417	TLR2/TLR1	2
BP	GO:0002429	immune response-activating cell surface receptor signaling pathway	0.03825489	0.026733056	MNDA/LYN/FCN1/FCER1G	4
BP	GO:0002757	immune response-activating signal transduction	0.03825489	0.026733056	MNDA/LYN/FCN1/FCER1G	4

BP	GO:0097530	granulocyte migration	0.041074486	0.028703429	TNFAIP6/FCER1G/CCL20	3
BP	GO:0050792	regulation of viral process	0.043667472	0.030515444	FCN1/CXCR4/SLPI	3
BP	GO:0030195	negative regulation of blood coagulation	0.044892214	0.031371311	SERPING1/CD9	2
BP	GO:0051250	negative regulation of lymphocyte activation	0.045524209	0.031812958	MNDA/LYN/SAMSN1	3
BP	GO:0043277	apoptotic cell clearance	0.045524209	0.031812958	FCN1/CD36	2
BP	GO:1900047	negative regulation of hemostasis	0.045524209	0.031812958	SERPING1/CD9	2
BP	GO:0002762	negative regulation of myeloid leukocyte differentiation	0.046900816	0.03277495	LYN/TNFAIP6	2
BP	GO:0002768	immune response-regulating cell surface receptor signaling pathway	0.048980523	0.034228279	MNDA/LYN/FCN1/FCER1G	4
BP	GO:0050819	negative regulation of coagulation	0.049678713	0.034716183	SERPING1/CD9	2
BP	GO:0038093	Fc receptor signaling pathway	0.050298558	0.035149341	LYN/FCER1G	2
BP	GO:1902991	regulation of amyloid precursor protein catabolic process	0.050298558	0.035149341	LYN/RTN1	2
BP	GO:0032635	interleukin-6 production	0.050298558	0.035149341	TLR2/TLR1/CD36	3
BP	GO:0032675	regulation of interleukin-6 production	0.050298558	0.035149341	TLR2/TLR1/CD36	3
BP	GO:0008347	glial cell migration	0.053079575	0.037092754	CD9/LAMB1	2
BP	GO:0022029	telencephalon cell migration	0.058187604	0.040662317	CXCR4/LAMB1	2
BP	GO:0002449	lymphocyte mediated immunity	0.05945731	0.041549605	SERPING1/FCER1G/CD1D/GZMB	4
BP	GO:0050777	negative regulation of immune response	0.060232408	0.042091255	LYN/SERPING1/SAMSN1	3
BP	GO:0090303	positive regulation of wound healing	0.060293617	0.042134028	CXCR4/CD36	2
BP	GO:0021885	forebrain cell migration	0.060293617	0.042134028	CXCR4/LAMB1	2
BP	GO:0032615	interleukin-12 production	0.060293617	0.042134028	TLR2/CD36	2
BP	GO:0032655	regulation of interleukin-12 production	0.060293617	0.042134028	TLR2/CD36	2
BP	GO:0032731	positive regulation of interleukin-1 beta production	0.060293617	0.042134028	MNDA/CD36	2
BP	GO:0070542	response to fatty acid	0.060293617	0.042134028	TLR2/CD36	2

BP	GO:0060760	positive regulation of response to cytokine stimulus	0.063589547	0.04443727	CXCR4/TLR2	2
BP	GO:0002695	negative regulation of leukocyte activation	0.063623091	0.044460712	MNDA/LYN/SAMSN1	3
BP	GO:0030888	regulation of B cell proliferation	0.066472023	0.046451585	MNDA/LYN	2
BP	GO:0042987	amyloid precursor protein catabolic process	0.067583759	0.047228482	LYN/RTN1	2
BP	GO:0050864	regulation of B cell activation	0.067583759	0.047228482	MNDA/LYN/SAMSN1	3
BP	GO:0071674	mononuclear cell migration	0.068924011	0.048165069	LYN/CXCR4/CCL20	3
CC	GO:0030139	endocytic vesicle	5.61E-05	3.95E-05	CTSS/CD163/LYN/TLR2/TLR1/NCF2/CD9/CD36	8
CC	GO:0031091	platelet alpha granule	6.87E-05	4.85E-05	SRGN/SERPING1/VWF/CD9/CD36	5
CC	GO:0101002	ficolin-1-rich granule	6.87E-05	4.85E-05	QPCT/CTSS/MNDA/FCN1/TNFAIP6/FCER1G	6
CC	GO:0030666	endocytic vesicle membrane	6.87E-05	4.85E-05	CD163/LYN/TLR2/TLR1/CD9/CD36	6
CC	GO:0034774	secretory granule lumen	6.87E-05	4.85E-05	QPCT/MNDA/FCN1/SRGN/SERPING1/VWF/SLPI	7
CC	GO:0009897	external side of plasma membrane	6.87E-05	4.85E-05	CD163/CLEC4A/FCN1/CXCR4/FCER1G/CD1D/CD9/CD36	8
CC	GO:0060205	cytoplasmic vesicle lumen	6.87E-05	4.85E-05	QPCT/MNDA/FCN1/SRGN/SERPING1/VWF/SLPI	7
CC	GO:0031983	vesicle lumen	6.87E-05	4.85E-05	QPCT/MNDA/FCN1/SRGN/SERPING1/VWF/SLPI	7
CC	GO:1904813	ficolin-1-rich granule lumen	7.48E-05	5.27E-05	QPCT/CTSS/MNDA/FCN1/TNFAIP6	5
CC	GO:0045335	phagocytic vesicle	0.000117388	8.28E-05	CTSS/TLR2/TLR1/NCF2/CD36	5
CC	GO:0062023	collagen-containing extracellular matrix	0.000307529	0.0002168	CTSS/VCAN/FCN1/SERPING1/VWF/LAMB1/SLPI	7
CC	GO:0030667	secretory granule membrane	0.000430572	0.000303542	FCGR3B/FCGR2A/TLR2/FCER1G/CD9/CD36	6
CC	GO:1904724	tertiary granule lumen	0.001789656	0.00126166	QPCT/CTSS/TNFAIP6	3
CC	GO:0044194	cytolytic granule	0.002610259	0.001840163	SRGN/GZMB	2

CC	GO:0031093	platelet alpha granule lumen	0.002671976	0.001883672	SRGN/SERPING1/VWF	3
CC	GO:0070820	tertiary granule	0.002671976	0.001883672	QPCT/CTSS/TNFAIP6/FCER1G	4
CC	GO:0031092	platelet alpha granule membrane	0.003467805	0.002444711	CD9/CD36	2
CC	GO:0045121	membrane raft	0.003467805	0.002444711	MS4A4A/LYN/TLR2/TLR1/CD36	5
CC	GO:0098857	membrane microdomain	0.003467805	0.002444711	MS4A4A/LYN/TLR2/TLR1/CD36	5
CC	GO:0005788	endoplasmic reticulum lumen	0.022747879	0.016036651	FSTL1/VCAN/SERPING1/LAMB1	4
CC	GO:0042581	specific granule	0.024258835	0.017101835	QPCT/SLPI/CD36	3
CC	GO:0098636	protein complex involved in cell adhesion	0.026802663	0.018895165	LYN/LAMB1	2
CC	GO:0005775	vacuolar lumen	0.028850494	0.020338832	CTSS/VCAN/MNDA	3
CC	GO:0035580	specific granule lumen	0.034573725	0.024373558	QPCT/SLPI	2
CC	GO:0030670	phagocytic vesicle membrane	0.050341729	0.035489582	TLR2/TLR1	2
MF	GO:0035325	Toll-like receptor binding	1.60E-06	1.05E-06	LY96/TLR2/TLR1/CD36	4
MF	GO:0019865	immunoglobulin binding	1.69E-05	1.11E-05	FCGR3B/FCGR2A/VWF/FCER1G	4
MF	GO:0038187	pattern recognition receptor activity	1.85E-05	1.21E-05	FCN1/LY96/TLR2/CD36	4
MF	GO:0071723	lipopeptide binding	4.60E-05	3.02E-05	TLR2/TLR1/CD1D	3
MF	GO:0019864	IgG binding	5.05E-05	3.32E-05	FCGR3B/FCGR2A/FCER1G	3
MF	GO:0005178	integrin binding	0.009338431	0.00613499	LYN/VWF/CD9/LAMB1	4
MF	GO:0003953	NAD+ nucleosidase activity	0.009338431	0.00613499	TLR2/TLR1	2
MF	GO:0050135	NAD(P)+ nucleosidase activity	0.009338431	0.00613499	TLR2/TLR1	2
MF	GO:0061809	NAD+ nucleotidase, cyclic ADP-ribose generating	0.009338431	0.00613499	TLR2/TLR1	2
MF	GO:0005540	hyaluronic acid binding	0.019108471	0.012553531	VCAN/TNFAIP6	2
MF	GO:0051861	glycolipid binding	0.023890535	0.015695163	LYN/FCGR3B	2
MF	GO:0005539	glycosaminoglycan binding	0.023890535	0.015695163	FSTL1/VCAN/TNFAIP6/TLR2	4
MF	GO:0001968	fibronectin binding	0.024515465	0.016105718	CTSS/TNFAIP6	2
MF	GO:0001530	lipopolysaccharide binding	0.031798943	0.020890683	LY96/TLR2	2
MF	GO:0016799	hydrolase activity, hydrolyzing N-glycosyl	0.031798943	0.020890683	TLR2/TLR1	2

		compounds				
MF	GO:0005044	scavenger receptor activity	0.044376863	0.029153893	CD163/CD36	2
MF	GO:0015026	coreceptor activity	0.044376863	0.029153893	LY96/CXCR4	2
MF	GO:0042277	peptide binding	0.051781819	0.034018665	TLR2/TLR1/CD1D/CD36	4
MF	GO:0005201	extracellular matrix structural constituent	0.052930961	0.034773607	VCAN/VWF/LAMB1	3
MF	GO:0004866	endopeptidase inhibitor activity	0.056057767	0.036827798	SERPING1/CSTA/SLPI	3
MF	GO:0030414	peptidase inhibitor activity	0.059245755	0.038922183	SERPING1/CSTA/SLPI	3
MF	GO:0061135	endopeptidase regulator activity	0.060780577	0.039930502	SERPING1/CSTA/SLPI	3
MF	GO:0097110	scaffold protein binding	0.060780577	0.039930502	CD163/LYN	2
MF	GO:0005518	collagen binding	0.063432695	0.041672842	CTSS/VWF	2

Abbreviations: BP, Biological Process; CC, Cell Component; MF, Molecular Function.

KEGG enrichment analysis results for differentially expressed genes

ID	Description	p.adjust	geneID	Count
hsa04145	Phagosome	0.000215889	CTSS/FCGR3B/FCGR2A/TLR2/NCF2/CD36	6
hsa05152	Tuberculosis	0.000287877	CTSS/FCGR3B/FCGR2A/TLR2/TLR1/FCER1G	6
hsa05140	Leishmaniasis	0.0014597	FCGR3B/FCGR2A/TLR2/NCF2	4
hsa04613	Neutrophil extracellular trap formation	0.002936414	FCGR3B/FCGR2A/TLR2/NCF2/VWF	5
hsa05417	Lipid and atherosclerosis	0.004191787	LYN/LY96/TLR2/NCF2/CD36	5
hsa04611	Platelet activation	0.004645074	LYN/FCGR2A/VWF/FCER1G	4
hsa04512	ECM-receptor interaction	0.019622491	VWF/LAMB1/CD36	3
hsa04666	Fc gamma R-mediated phagocytosis	0.019622491	LYN/FCGR3B/FCGR2A	3
hsa04640	Hematopoietic cell lineage	0.019622491	CD1D/CD9/CD36	3
hsa05146	Amoebiasis	0.019622491	TLR2/CD1D/LAMB1	3
hsa04064	NF-kappa B signaling pathway	0.019622491	LYN/LY96/BCL2A1	3
hsa04620	Toll-like receptor signaling pathway	0.019622491	LY96/TLR2/TLR1	3
hsa05145	Toxoplasmosis	0.022348725	LY96/TLR2/LAMB1	3
hsa04380	Osteoclast differentiation	0.03020781	FCGR3B/FCGR2A/NCF2	3
hsa04650	Natural killer cell mediated cytotoxicity	0.030722857	FCGR3B/FCER1G/GZMB	3
hsa04210	Apoptosis	0.031298495	CTSS/BCL2A1/GZMB	3
hsa05144	Malaria	0.041147196	TLR2/CD36	2
hsa04664	Fc epsilon RI signaling pathway	0.067557444	LYN/FCER1G	2
hsa04062	Chemokine signaling pathway	0.067557444	LYN/CXCR4/CCL20	3
